# Trientine induced colitis during therapy for Wilson disease: a case report and review of the literature

**DOI:** 10.1186/s40360-015-0031-z

**Published:** 2015-11-20

**Authors:** Salih Boga, Dhanpat Jain, Michael L. Schilsky

**Affiliations:** Division of Digestive Diseases and Section of Transplantation and Immunology, Department of Medicine and Surgery, Yale University School of Medicine, Yale University Medical Center, 333 Cedar Street, LMP 1080, New Haven, CT 06520 USA; Department of Pathology, Yale University School of Medicine, 310 Cedar Street, P.O. Box 208023, New Haven, CT 06520-8023 USA

**Keywords:** Trientine, Colitis, Wilson disease

## Abstract

**Background:**

Wilson disease (WD) is an autosomal recessive disorder of human copper metabolism characterized by copper accumulation in the liver due to impaired excretion of copper into the bile. Brain accumulation of copper may cause neuropsychiatric symptoms. Trientine (triethylenetetramine dihydrochloride) is a copper-chelating agent used to treat patients with WD. Trientine has been considered an option for initial treatment and maintentance therapy of WD due to its safety profile.

**Case presentation:**

A 40 year old female with a recent diagnosis of WD was started on treatment with trientine for her WD. Within one month she developed profound bloody diarrhea unresponsive to medical treatment. Trientine was discontinued and a colonoscopy with biopsy showed moderately active ileitis and moderate to severe pancolitis, consistent with a drug induced mucosal injury. The colitis improved immediately upon withdrawal of trientine, and recurred when medication was rechallenged because of worsened WD symptoms. After second compulsory discontinuation of trientine, she remained on zinc therapy for her WD and her colitis resolved by time.

**Conclusion:**

Drug induced colitis is a very rare side effect of trientine. Although trientine therapy is well tolerated and less side effects are reported with this medication than penicillamine, colitis can occur during trientine treatment. Zinc therapy may be an effective alternative for treatment of WD in patients experiencing side effects from chelation therapy.

## Background

Wilson disease (WD) is an autosomal recessive disorder of copper metabolism with prevalence of ~1:30,000. Untreated, the disease leads to progressive hepatic failure or debilitating neuropsychiatric symptoms [1]. Drug reactions due to d- Penicillamine, the first oral copper chelating agent, led to the development of triethylenetetramine dihydrochloride (trientine). The first reported use of trientine was by Walshe in 1969 to treat a child who developed penicillamine-induced nephrotoxicity [2]. Since, trientine has been used to treat WD with rare reported side effects described in case reports or small case series. Here we report on a WD patient on trientine therapy who experienced colitis and discuss her management and treatment.

## Case presentation

A 40 year old female with a recent diagnosis of WD with predominant neuropsychiatric symptoms was referred. She was initially treated by her primary care physician for a month with escitalopram, 5 mg daily. She noted increased agitation, so escitalopram was discontinued. Over the next 8 months, she did not complain of depression, however developed perioral and fingertip paresthesias, gait unsteadiness and slowed speech. Further testing included electromyography, and spinal and cranial magnetic resonance imaging, all reported as normal. Additional testing leading to the diagnosis of WD included a ceruloplasmin of 3 mg/dl (14–47.8), serum copper of 0.35 μg/dl (0.75–1.45) and a 24 h urıne copper excretion of 149 μg (15–60). Kayser-Fleisher rings were seen on ophthalmological evaluation. She was started on treatment for WD with trientine 250 mg three times daily. Within one month she developed profound bloody diarrhea with negative cultures for enteric pathogens, including clostridium difficile, and was unresponsive to immodium, ciprofloxacin, and lomotil. Trientine was discontinued and a colonoscopy with biopsy was performed. Biopsies showed moderately active ileitis and moderate to severe pancolitis. The changes were diffuse with an increase in lamina propria lymphoplasmacytic infiltration, diffuse injury to crypts with marked mucin depletion and extensive cryptitis. The crypt distortion was mild and there was no granulomatous inflammation or paneth cell metaplasia. The histologic changes were suggestive of active colitis, but were etiologically non-specific. Given her history, the colonic changes were consistent with a drug induced mucosal injury.

She was started on prednisone for the colitis. Three days later she became severely manic with psychotic features, prompting psychiatry consultation. She was treated symptomatically with atypical antipsychotics to which she experienced side effects at very low doses (Olanzapine 2.5 mg, Quetiapine 50 mg). She was maintained on zinc, 50 mg, three times daily for her WD while the prednisone was tapered off. Her colitis symptoms resolved. On follow-up sigmoidoscopy, colitis was resolved.

**Fig. 1:**
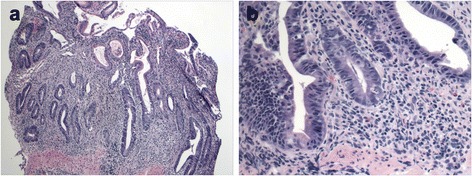
**a**. Low magnification of the biopsy showing diffuse infiltration of the colonic lamina propria by lymphoplasmacytic inflammatory infiltrate, slight crypt distortion, focal crypt shortening, and crypt abscess. **b**. Higher magnification showing mucin depletion of the crypt epithelium and reactive changes. Cryptitis can also be easily seen at this magnification

After an initial improvement, her psychosis worsened and she required hospitalization. Due to neuropsychiatric symptoms, trientine was restarted in addition to the zinc for her WD. She was transferred to the medical floor after several days due to recurrent colitis with multiple grossly bloody bowel movements. Trientine was discontinued and she was maintained on zinc therapy for her WD. Evaluation for other causes of colitis included negative blood and stool cultures, including C. Difficile, campylobacter, salmonella and norovirus. She also had no antibodies to Saccharomyces cerevisiae, normal Ig A and Ig G levels, and ANCA and p-ANCA were negative. Repeat colonoscopy showed moderate to severe active colitis and proctitis, and the degree of colitis was significantly more severe than on prior biopsies. There was extensive acute inflammation with cryptitis and crypt abscess formation and multiple foci of fresh and healing ulceration. Occasional branching crypts and focal crypt shortening was noted. Similar to the prior biopsies, granulomas, pseudopyloric metaplasia or paneth cell metaplasia were not seen (Fig. 1a&1b). While the histologic differential diagnosis included prolonged infectious, self-limited type colitis and inflammatory bowel disease. Given the clinical features, drug induced colitis was favored. With time and continuation of her zinc therapy for her WD, her colitis resolved.

Her psychiatric symptoms also improved, and nine months after the onset of psychiatric symptoms she was symptoms-free, having returned to work and baseline social activities. Four years later she continues in remission psychiatrically with stable liver function. She remains on zinc maintenance therapy for her WD.

## Discussion

Medical treatment regimens for WD include copper chelators and zinc salts. Chelating agents are generally first line therapy for symptomatic patients because of their higher decoppering potential compared to zinc salts that indirectly reduce copper by blocking intestinal absorption. D-penicillamine, the first oral copper chelating agent [3], is commonly used worldwide. However intolerance and serious side effects such as immune complex nephritis, nephrotic syndrome, systemic lupus erythematosus and bone marrow suppression led to the use of trientine. [2]. Trientine is an effective initial treatment and maintentance therapy for WD [4]. Due to its better safety profile, trientine was included as an option for first line and maintenance therapy for WD in American Association for the Study of Liver Diseases (AASLD) and European Association for the Study of Liver Diseases (EASL) guidelines [5, 6]. Rare side effects reported for trientine include iron deficiency, systemic lupus erythematosus, dystonia, muscular spasm and myasthenia gravis [7]. Only 6–18 % of orally administered trientine is systemically absorbed [8].

Effects of trientine on the colon were first noted by Dahlman et al. in 1995, profiling two WD patients that experienced colitis while on trientine [9]. Their first patient had experienced side effects with penicillamine, developing uveitis, proteinuria and ANA positivity, and was changed to trientine. Endoscopic evidence of duodenitis and colitis was seen four months after exposure to trientine. Although trientine was initially withdrawn it was later restarted along with steroid. The steroid was eventually withdrawn, and the patient remained on trientine without diarrhea or recurrence of colitis.

The second WD patient was cirrhotic and was initially started on trientine. Diarrhea developed that ceased with the withdrawal of a neuroleptic drug, and trientine was continued. Months later, when symptoms started with a gastroenteritis seen in the whole family, the patient developed bloody diarrhea and had colonoscopic evidence of colitis. After withdrawal of trientine, colitis recurred when penicillamine was administered, suggesting an underlying inflammatory bowel disease.

In our WD patient, trientine was chosen as initial treatment. Colitis developed within a month of exposure to the drug, earlier than in the cases reported above. Her symptoms improved immediately upon withdrawal of trientine, and colitis recurred with rechallenge to this medication. The colitis was visible on colonoscopy from the rectum to the transverse colon. Histologic features included cryptitis, crypt abscesses and multiple foci of fresh and healing ulcerations. The histologic changes are not specific, and could be seen in infectious colitis and evolving inflammatory bowel disease. The lack of well-developed features of chronicity like established crypt architectural distortion and paneth and the timing of the colitis were most helpful in making a diagnosis of trientine colitis, with features similar to those reported in previous cases [9]. The pathophysiological mechanism for the development of colitis is unknown. As noted, there is poor intestinal absorption of trientine from the gut, and the trigger for developing colitis may be a local reaction with the colonic epithelium rather than of autoimmune origin, especially since the reaction was limited to the time of medication exposure.

Treatment with a chelating agent was warranted in our patient with symptomatic disease with neuropsychiatric features dominating the clinical presentation. Due to the continued severe neuropsychiatric symptoms, the re-challenge with trientine was justified. It is worth notion that the steroids required for treatment of trientine induced colitis complicated the differential diagnosis of her psychosis. However, the duration of psychosis (months after steroids were discontinued) and the lack of exacerbation with another challenge of prednisone led to the working diagnosis of psychosis secondary to WD rather than steroid induced psychosis. After the withdrawal of trientine, the patient was effectively treated with zinc therapy. Zinc salts induce intestinal epithelium to produce cytosolic metallothioneins that effectively sequester copper and block new copper absorption. While zinc is indicated for maintenance therapy for WD or initial treatment of asymptomatic WD patients, zinc was used for first line treatment of some patients with neurological WD [10]. Though treatment with zinc can sometimes cause gastric side effects [11] and the achievement of a negative copper balance is slower with zinc, in patients such as ours, zinc therapy was effective over time. Since our patient had psychiatric symptoms, she was treated for these independent of the WD, and was able to withdraw psychiatric therapy over 1–2 years time.

At the time of the reporting of the prior cases of trientine colitis, zinc therapy was a less well accepted alternative treatment of WD. Therefore the re-trial of the drug and attempts at steroid desensitization were warranted. Given the numerous reports demonstrating the efficacy of zinc therapy for WD, there would no longer be justification for desensitization to trientine or d-penicillamine in intolerant patients.

## Conclusion

Because WD is a rare disease and trientine is an orphan drug, controlled trials that demonstrate the true incidence of medication side effects with adequate sample size sufficient to be graded as high-quality studies are not available. It is our experience that trientine therapy is well tolerated, and less side effects are reported with this medication than penicillamine. However drug induced colitis can occur during trientine treatment, and clinicians need to be aware of this entity if their patient develops a diarrheal illness while on this therapy. Re-challenge with trientine is not warranted unless there is a known intolerance to zinc therapy. Zinc therapy may be an effective alternative for treatment of WD in patients experiencing side effects from chelation therapy.

## Consent

Written informed consent was obtained from the patient for publication of this case report and any accompanying images. A copy of the written consent is available for review by the Editor of this journal.

## Abbreviations

WD: Wilson disease
ANCA: Anti-neutrophil cytoplasmic antibody
p-ANCA: Perinuclear anti-neutrophil cytoplasmic antibody
ANA: Anti-nuclear antibody
